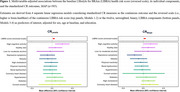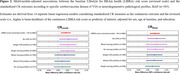# Associations of the LIfestyle for BRAin health index with Cognitive Resilience to neuropathology

**DOI:** 10.1002/alz70860_097995

**Published:** 2025-12-23

**Authors:** Maude Wagner, Puja Agarwal, Klodian Dhana, Sonal Agrawal, Sue E. Leurgans, David A. A. Bennett, Julie A Schneider, Melissa Lamar

**Affiliations:** ^1^ Rush Alzheimer's Disease Center, Rush University Medical Center, Chicago, IL, USA; ^2^ Rush Alzheimer's Disease Center, Chicago, IL, USA

## Abstract

**Background:**

Cognitive resilience (CR) can be defined as the continuum of better‐ through worse‐than‐expected cognition, given the burdens of neuropathology. Whether modifiable lifestyle and health‐related risk factors may promote CR remains unclear.

**Method:**

We selected 585 decedents (88 years at death; 71% female) from the Rush Memory and Aging Project, who completed at least two cognitive evaluations and had no missing data for the cerebrovascular disease (CVD) pathologies (atherosclerosis, arteriolosclerosis, macroinfarcts, microinfarcts, cerebral amyloid angiopathy) and neurodegenerative pathologies (AD, TDP‐43, neocortical Lewy bodies, hippocampal sclerosis) systematically examined at autopsy. Participants had no dementia at the initial assessment of the LIfestyle for BRAin health (LIBRA) index, a validated weighted risk score that uniquely adds the deleterious effects of twelve modifiable components, including unhealthy lifestyle, poor cardiometabolic health, and depression. Using linear mixed‐effects models adjusted for demographics and neuropathologies, we first estimated two complementary CR measures, with positive values reflecting higher cognitive levels (CR_Levels_) and slower cognitive decline (CR_Slope_) than expected before death, given neuropathology. Then, using separate linear regression models adjusted for demographics, we examined the associations of the LIBRA index and its components with the two continuous CR outcomes.

**Result:**

Lower LIBRA risk scores at baseline, denoting reduced dementia risk, were linearly associated with higher CR_Levels_ (mean difference [MD] for 1‐point lower in LIBRA=0.04; 95%CI=0.02,0.07) and higher CR_Slope_ (MD=0.04; 95%CI=0.01,0.06) (Figure 1). When examining the individual LIBRA components simultaneously, cognitively stimulating activities showed the strongest associations with both CR measures (Figure 1). Lastly, in stratified analyses considering six specific neuropathological profiles (no, single, mixed CVD; no, single, mixed neurodegenerative), we found that lower LIBRA scores were associated with higher CR_Levels_ in participants without CVD (MD=0.06; 95%CI=0.01,0.10), in those with a single CVD (MD=0.05; 95%CI=0.01,0.10) and in those with a single neurodegenerative pathology (MD=0.05; 95%CI=0.03,0.09) (Figure 2). No associations were found for CR_Slope._

**Conclusion:**

These findings support that prevention programs targeting lifestyle modifications and management of other health‐related factors could help to preserve cognitive health in older adults by promoting cognitive resilience to neuropathology. The findings also suggest that older adults with fewer neuropathological insults may see larger improvements in resilience.